# The ribosomal RNA synthesis ratio biomarker in *Mycobacterium ulcerans* for drug activity evaluation

**DOI:** 10.1186/s40249-026-01449-2

**Published:** 2026-05-08

**Authors:** Juan Calvet-Seral, Emma Sáez-López, Patricio R. López-Expósito, Santiago Ferrer-Bazaga, Juan José Vaquero, Santiago Ramón-García, Alfonso Mendoza-Losana

**Affiliations:** 1 Biomedical Sciences and Engineering Laboratory, Departamento de Bioingeniería, Universidad Carlos III de Madrid, Madrid, Spain; 2 Department of Microbiology, Pediatrics, Radiology and Public Health, Faculty of Medicine, University of Zaragoza, Zaragoza, Spain; 3 Spanish Network for Research on Respiratory Diseases (CIBERES), Carlos III Health Institute, Madrid, Spain; 4 Instituto de Investigación Sanitaria Gregorio Marañón, Madrid, Spain; 5 Research & Development Agency of Aragón Foundation (Fundación ARAID), Zaragoza, Spain

**Keywords:** Ribosomal RNA synthesis ratio, Buruli ulcer, Mycobacterium ulcerans, Biomarker, Treatment-shortening, Mycobacteria, Drug combinations

## Abstract

Buruli ulcer (BU), a neglected tropical disease caused by *Mycobacterium ulcerans* (*Mul*), is treated with an 8-week regimen of rifampicin (RIF) and clarithromycin (CLA). Clinical trials are currently evaluating amoxicillin/clavulanate (AMX/CLV) co-administration to reduce treatment duration. However, conventional methods for assessing in vitro drug efficacy against *Mul*, like colony-forming units (CFUs), are slow and cumbersome. The ribosomal RNA synthesis ratio (RS-ratio) measures ribosome biogenesis and serves as a proxy of metabolic activity. While it is a promising predictive biomarker for treatment shortening in tuberculosis, its application in *Mul* remains unexplored. Here, we evaluated the RS-ratio for *Mul* drug activity assessment through RNA extractions from time-kill assays using RIF, CLA, and AMX/CLV, alone or in combinations. RIF + AMX/CLV-containing combinations produced a potent, rapid RS-ratio reduction, decreasing from a baseline of ≈2000 to ≈200 as early as day 3, and reaching their maximal inhibition (≈50–60) between days 7 and 10. Notably, this metabolic decline preceded the CFUs and luminescence drops observed in prior studies. Interestingly, the RS-ratio detected a metabolic recovery between days 14 and 28 (≈400), suggesting remaining bacterial viability, a phenomenon not observed by CFUs or luminescence. In summary, this is the first report using the RS-ratio to evaluate antibiotic activity against *Mul*. Our findings validate the RS-ratio as a molecular tool for assessing the sterilizing potential of new regimens to inform future research and clinical trial designs for the treatment of BU. Our results support the RIF + CLA + AMX/CLV regimen selection for BU treatment shortening in the BLMs4BU clinical trials (NCT05169554, PACTR202209521256638).

**Graphical Abstract:**

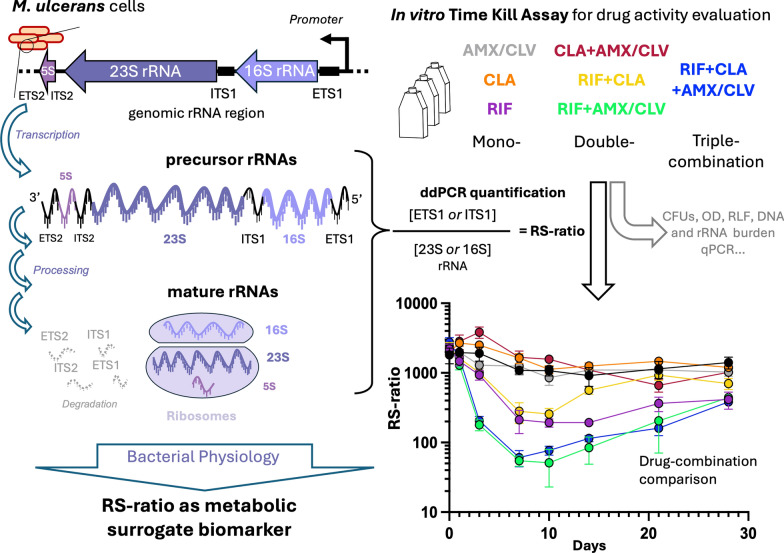

## Introduction

*Mycobacterium ulcerans* (*Mul*) is the causative agent of Buruli ulcer (BU), a debilitating skin-neglected tropical disease (NTD) that predominantly affects communities in rural areas with limited access to healthcare services. Despite its low mortality rate, BU can lead to significant morbidity due to the destruction of skin, soft tissues, and even bones. In the absence of early treatment, this may result in irreversible disfigurement and disability with high socio-economic burden and stigma. BU cases have been reported in 33 countries, with over 80% of global cases in the African Region, where nearly 50% of the affected individuals are children under the age of 15 [1]. The current World Healt Organization (WHO)-recommended antibiotic treatment involves an 8-week daily regimen of rifampicin (RIF) and clarithromycin (CLA). Treatment also includes extensive wound care, sometimes surgical intervention, and physiotherapy rehabilitation of movement limitations and disability, with lesion healing potentially lasting for several months. Treatment compliance can be difficult due to socioeconomic factors. A shortened and highly effective regimen would improve the care of BU patients and result in cost reduction. Consequently, the WHO requires evaluating promising medicines to provide new treatment options, including shortening the duration of treatment, as outlined in the WHO NTD Road Map 2021–2030 [1, 2].

In this context, using a drug repurposing approach, Arenaz et al. [3] investigated the in vitro effects of combining various β-lactam antibiotics with RIF and/or CLA. By utilizing checkerboard assays, they demonstrated a strong synergistic effect of amoxicillin/clavulanate (AMX/CLV) with RIF, evidenced by a substantial reduction in the Minimum Inhibitory Concentration (MIC) of RIF and CLA at a fixed time point; these promising findings were subsequently corroborated by in vitro time-kill assays (TKA) [4], which paved the way for the BLMs4BU clinical trial currently underway in several African countries (https://blms4bu.org/) (NCT05169554, PACTR202209521256638) [5, 6]. In addition, Saez et al. [4] conducted a comparative analysis of different in vitro readout measurements; this analysis demonstrated the value of a wide variety of biomarkers in BU research, correlating colony-forming units (CFUs) (the in vitro gold standard method for quantifying bacterial load) with optical density at 600 nm, luminescence production, quantification of the IS*2404* DNA by quantitative PCR (qPCR), and the 16S rRNA burden by reverse transcriptase qPCR [4]. This comparison was motivated by the arduous nature of CFU determination for *Mul*, which necessitates manipulation in biosafety level 3 (BSL3) laboratories and is hampered by the bacterium's slow growth rate on agar, often requiring from 1 to 3 months for visible colonies to appear. Furthermore, the study identified 16S rRNA burden quantification and luminescence measurements as having the strongest correlation with each other and a satisfactory correlation with CFU determination, with luminescence being the most cost-effective method. However, beyond conventional phenotypic metrics such as CFU quantification, there is an emerging consensus on the translational significance of evaluating the molecular metabolic state of bacteria as a proxy for the sterilizing activity of drug regimens. Sterilizing drugs, which can eradicate all viable bacterial organisms, are imperative for achieving effective shortening treatment regimens.

Ribosomal RNA (rRNA) constitutes a major component of the ribosome, comprising 80–85% of the total RNA present in cells. Its synthesis is subject to stringent regulation as it is a critical rate-limiting step for ribosome biogenesis and, consequently, protein synthesis and cell growth. The ribosomal RNA synthesis ratio (RS-ratio) has recently been described as a novel pharmacodynamic marker used to assess the effectiveness of drugs against *Mycobacterium tuberculosis* (*Mtb*) [7], which belongs to the same genus as *Mul*. The RS-ratio is a molecular assay that quantifies the abundance of a rapidly processed spacer region present in the precursor polycistronic rRNA (immature) relative to the stable final rRNA (mature) sequences. The ratio between these two types of rRNA (immature *vs.* mature) provides a measure of how actively the bacteria are synthesizing new ribosomes, which is a proxy for their physiological activity and replication rate. This provides different information than conventional markers, such as CFU or rRNA burden by 16S rRNA quantification.

In *Mtb,* it has been demonstrated that in vitro monotherapies of RIF and bedaquiline (known to be the primary sterilizing agents in tuberculosis (TB) treatment) induce a significantly stronger and faster reduction in the RS-ratio than isoniazid, streptomycin, and ethambutol, which reduce CFU counts more effectively, but without substantially reducing the RS-ratio. In fact, drug combinations that induce faster and stronger reductions in the RS-ratio correlate with improved treatment outcomes and reduced relapse rates in murine models of TB [7]. Thus, the RS-ratio is highly sensitive to the sterilizing effect of treatments, being a promising biomarker for the evaluation of the treatment-shortening potential of new drugs and regimens in development. However, despite its potential, the application of the RS-ratio as a tool for drug activity evaluation has primarily focused on *Mtb*, with limited exploration in other mycobacterial species, as evidenced by the lack of reports in the case of *Mul*.

In this study, we describe for the first time the in vitro development and application of the RS-ratio biomarker to *Mul* to evaluate the in vitro activity of RIF, CLA, and AMX/CLV and their combinations, currently under clinical investigation for BU treatment shortening. The results underpin the benefits of the RS-ratio as an in vitro biomarker and point to its relevance for future use at the clinical level.

## Materials and methods

### Genetic material

For this proof-of-concept study, RNA samples from *Mul* were retrospectively obtained from TKA samples of the biomarker benchmarking described by Saez et al*.* [4]. In that study, the *Mul* isolate ITM 000932 was exposed to RIF 0.025 µg/ml (1/2 × MIC), CLA 0.0625 µg/ml (1 × MIC), and AMX 0.125 µg/ml combined with CLV 5 µg/ml (1 × MIC AMX in the presence of CLV). Drugs were administered alone or in combination in single independent biological replicates. Additionally, RNA extracts from the *Mtb* H37Ra and *Mycobacterium smegmatis* (*Msm*) mc^2^155 strains were used to test amplicon design specificities. Complementary DNA (cDNA) was synthesized using the SuperScript IV VILO Master Mix (Invitrogen, Carlsbad, USA) in a 10 µl reaction volume, using 5 µl of RNA (50 ng–1 µg) as template. Reverse transcription conditions were 25 °C for 10 min, followed by 50 °C for 15 min in a C1000 Touch Thermal Cycler (Bio-Rad Laboratories, Inc., Hercules, USA).

### Oligonucleotide design for *M. ulcerans* rRNA targets

**Fig. 1:**
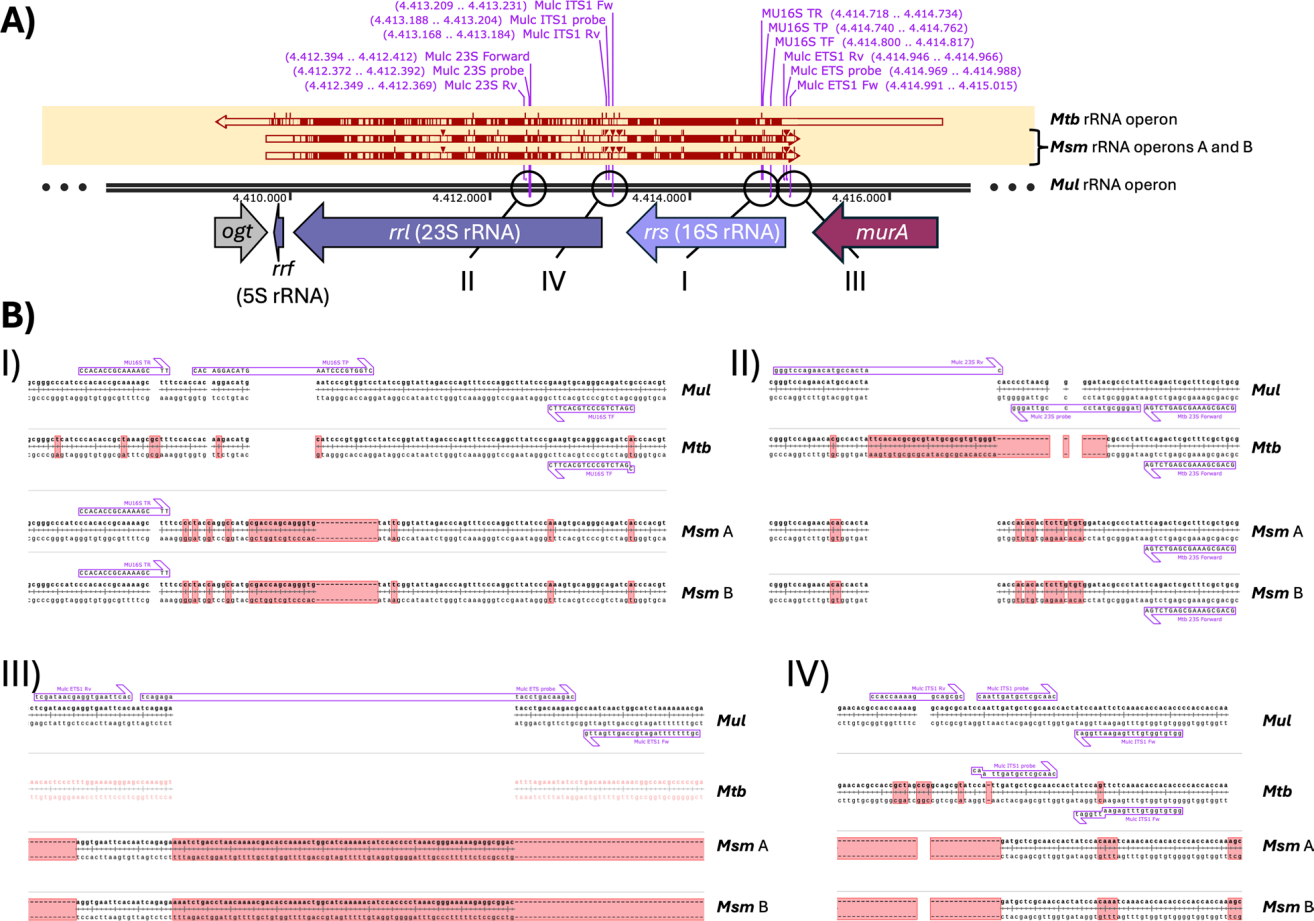
**A** Localization and **B** Specific alignments compared with *Mtb* and *Msm* rRNA operons of the **I** 16S, **II** 23S, **III** ETS1, and **IV** ITS1 primers-probe sets synthesized to calculate the RS-ratio in *Mul*. *Mtb M. tuberculosis*, *Mul M. ulcerans*, *Msm M. smegmatis*. *RS-ratio rRNA synthesis ratio*

Primers-probe sets for the quantification of the external transcribed spacer 1 (ETS1), internal transcribed spacer 1 (ITS1), and 23S rRNA of *Mul* were designed to target homologous regions to those used for RS-ratio calculations in *Mtb* [7] (Fig. 1A). These sequences were designed, ensuring species specificity, aligning *Mul*, *Mtb,* and *Msm* rRNA operon regions using SnapGene v8.0.3 software (Dotmatics, Boston, USA). To ensure high specificity and prevent cross-amplification, at least one oligonucleotide within each primer and probe set was designed to not align with the rRNA operons from published sequences of *Mtb* (NCBI accession number NC_018143) and *Msm* (NCBI accession number NC_008596) (Fig. 1B). Specificity was confirmed using NCBI Nucleotide BLAST (National Center for Biotechnology Information, Bethesda, USA). Additionally, we used the primers-probe sequences for detecting the *Mul* 16S rRNA previously published [8], which already met the specificity criterion (Fig. 1BI). Fluorophore probes were selected to allow differential detection in the QX600 system (Bio-Rad Laboratories, Inc., Hercules, USA) channels: Channel 1 for ETS1, Channel 2 for 23S rRNA, Channel 3 for 16S rRNA, and Channel 5 for ITS1. Bio-Rad Laboratories synthesized the ETS1, 23S, and 16S primers-probe sets, and the ITS1 primers-probe set was synthesized by CerTest (CerTest Biotec, S.L., San Mateo de Gállego, España) (Table 1).

**Table 1:** Primers and probes used to calculate the RS-ratio for *Mycobacterium ulcerans*

Target		Sequence	5´mod	3' mod	References
ETS1 *Mul*	Fw	CGTTTTTTTAGATGCCAGTTGATTG	FAM	Iowa Black	This work
	Probe	TCAGAGATACCTGACAAGAC			
	Rv	TCGATAACGAGGTGAATTCAC			
23S rRNA *Mul*	Fw	GCAGCGAAAGCGAGTCTGA	HEX	Iowa Black	This work
	Probe	TAGGGCGTATCCCCGTTAGGG			
	Rv	GGGTCCAGAACATGCCACTAC			
16S rRNA *Mul*	Fw	CGATCTGCCCTGCACTTC	Cy5	Iowa Black	Beissner et al. [8]
	Probe	CACAGGACATGAATCCCGTGGTC			
	Rv	CCACACCGCAAAAGCTT			
ITS *Mul*	Fw	GGTGTGGTGTTTGAGAATTGGAT	ROX	BHQ2	This work
	Probe	CAATTGATGCTCGCAAC			
	Rv	CCACCAAAAGGCAGCGC			

*Fw* forward, *Rv* reverse, *ETS1* external transcribed spacer 1, *ITS1* internal transcribed spacer 1, *cDNA FAM* 6-Carboxyfluorescein, *HEX* Hexachlorofluorescein, *Cy5* Cyanine-5, *ROX* Rhodamine X, *BHQ2* Black Hole Quencher^®^-2. *RS-ratio* rRNA synthesis ratio

### Droplet digital PCR and RS-ratio determination for *M. ulcerans*

Primers and probe sets for droplet digital PCR (ddPCR) quantification were used in a single multiplexed reaction at Bio-Rad's recommended concentrations (900 nmol/L for primers and 250 nmol/L for probes) with ddPCR SuperMix for Probes (no dUTP) (Bio-Rad Laboratories, Inc., Hercules, USA), and 2 µl of diluted cDNA as template. The dilution tested was sample-specific and ranged from 10^–4^ to 10^–9^ to enable accurate quantification of all four targets. Thermocycling conditions included an initial denaturation step at 95 °C for 10 min, followed by 40 cycles of denaturation at 96 °C for 30 s and annealing/extension at 60 °C for 90 s in a C1000 Touch Thermal Cycler. A signal stabilization step was performed at 98 °C for 10 min, followed by a final hold at 4 °C for at least 30 min before droplet reading and quantification in the QX600 system. The temperature ramp rate was set at 2 °C/s for all steps. The number of copies for each target was determined from the partitioned droplets using the QuantaSoft v2.1 software (Bio-Rad Laboratories, Inc., Hercules, USA). The RS-ratio was calculated as the ratio of the copy number of the precursor rRNA (ETS1 or ITS1) to the copy number of the total rRNA (23S or 16S), multiplied by 10^4^, as previously described [7]. The RS-ratio values in this study represent the mean and standard deviation from technical replicates using different cDNA dilutions. These data are derived from single independent biological replicates, consistent with the proof-of-concept design from the parent study [4]. Data were plotted using GraphPad Prism V10.4.0 (527) (GraphPad Software, San Diego, USA).

## Results

### Validation of specificity and ddPCR optimization for *M. ulcerans* RS-ratio determination

**Fig. 2:**
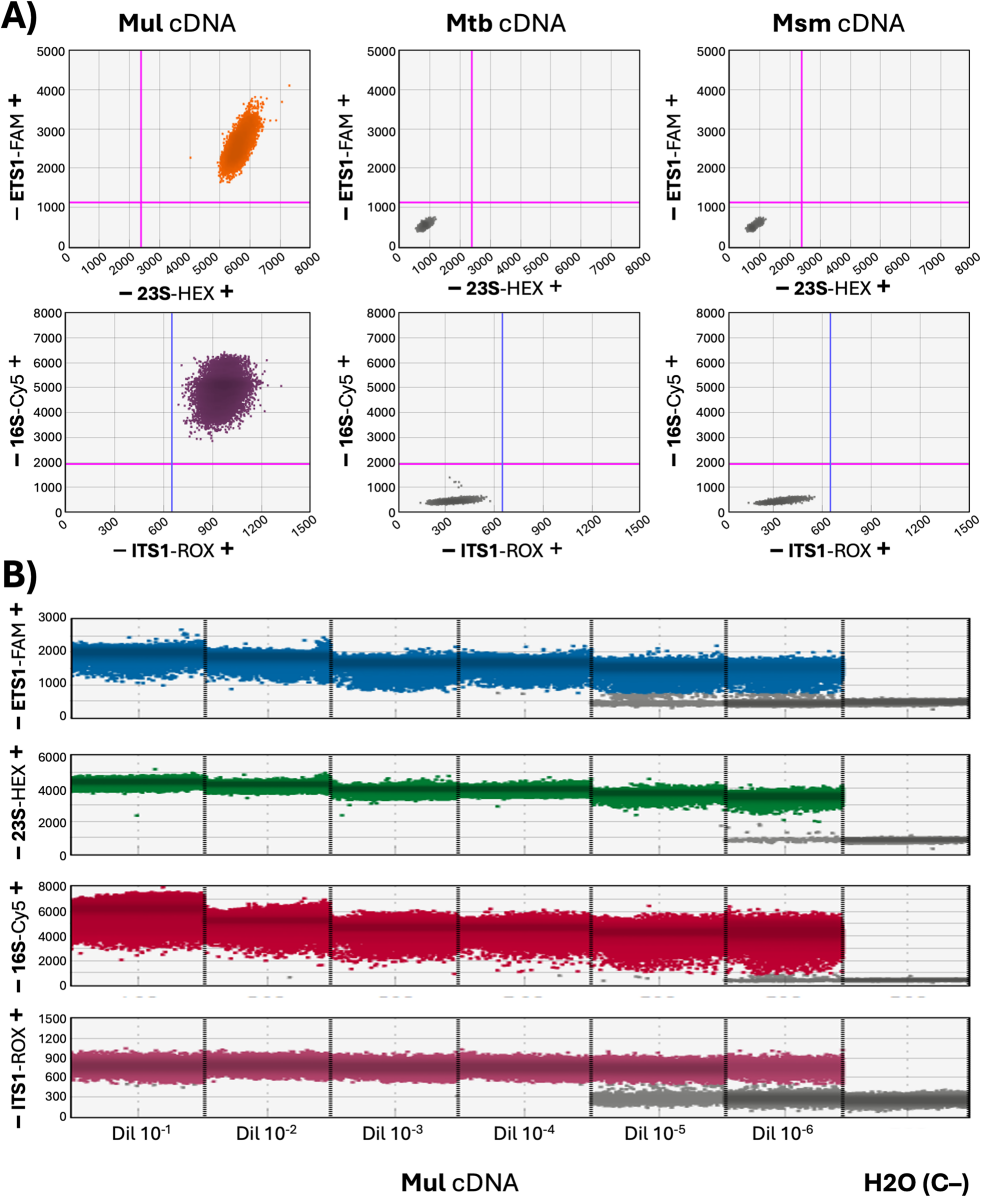
Multiplexed *Mul* ddPCR. **A** *Mul* ddPCR is species-specific. Quadruple positive droplets were obtained with high concentrations of *Mul* cDNA, whereas quadruple negative droplets were obtained with cDNA from *Mtb* or *Msm*. **B** *Mul* cDNA samples need to be diluted to allow target quantification. Several tenfold dilutions of the *Mul* cDNA were required to obtain different positive and negative droplet populations, allowing for the quantification of the different targets. *Mtb M. tuberculosis*, *Mul M. ulcerans*, *Msm M. smegmatis*, *ETS1* external transcribed spacer 1, *ITS1* internal transcribed spacer 1, *cDNA* complementary DNA, *FAM* 6-Carboxyfluorescein, *HEX* Hexachlorofluorescein, *Cy5* Cyanine-5, *ROX* Rhodamine X, *C-* negative control

To experimentally confirm their specificity, the newly synthesized oligonucleotide sets were subjected to a series of tests using a multiplexed ddPCR reaction with cDNA extracts from *Mul*, *Mtb*, and *Msm*. The *Mul* cDNA sample saturated the quantification when undiluted, resulting in 100% positive droplets for all four targets (ETS1, ITS1, 23S rRNA, and 16S rRNA). Crucially, no positive droplets were obtained from the cDNA samples of *Mtb* and *Msm*, thus confirming the high specificity of the designed probes and primers for *Mul* targets (Fig. 2A).

The RS-ratio was determined for the *Mul* ITM 000932 isolate longitudinally exposed to RIF, CLA, and AMX/CLV at 1/2 × , 1 × , and 1 × MIC values, respectively, both in monotherapy and in two- and three-way combinations [4]. To ensure accurate quantification and prevent saturation of the ddPCR reaction, each cDNA extract from the *Mul* TKA samples (3) was optimized for specific dilution, allowing for quantifiable droplet numbers for all four targets (ETS1, ITS1, 23S rRNA, and 16S rRNA) (Fig. 2B).

### Evaluation of dynamic range and selection of the optimal precursor/total rRNA pair

**Fig. 3:**
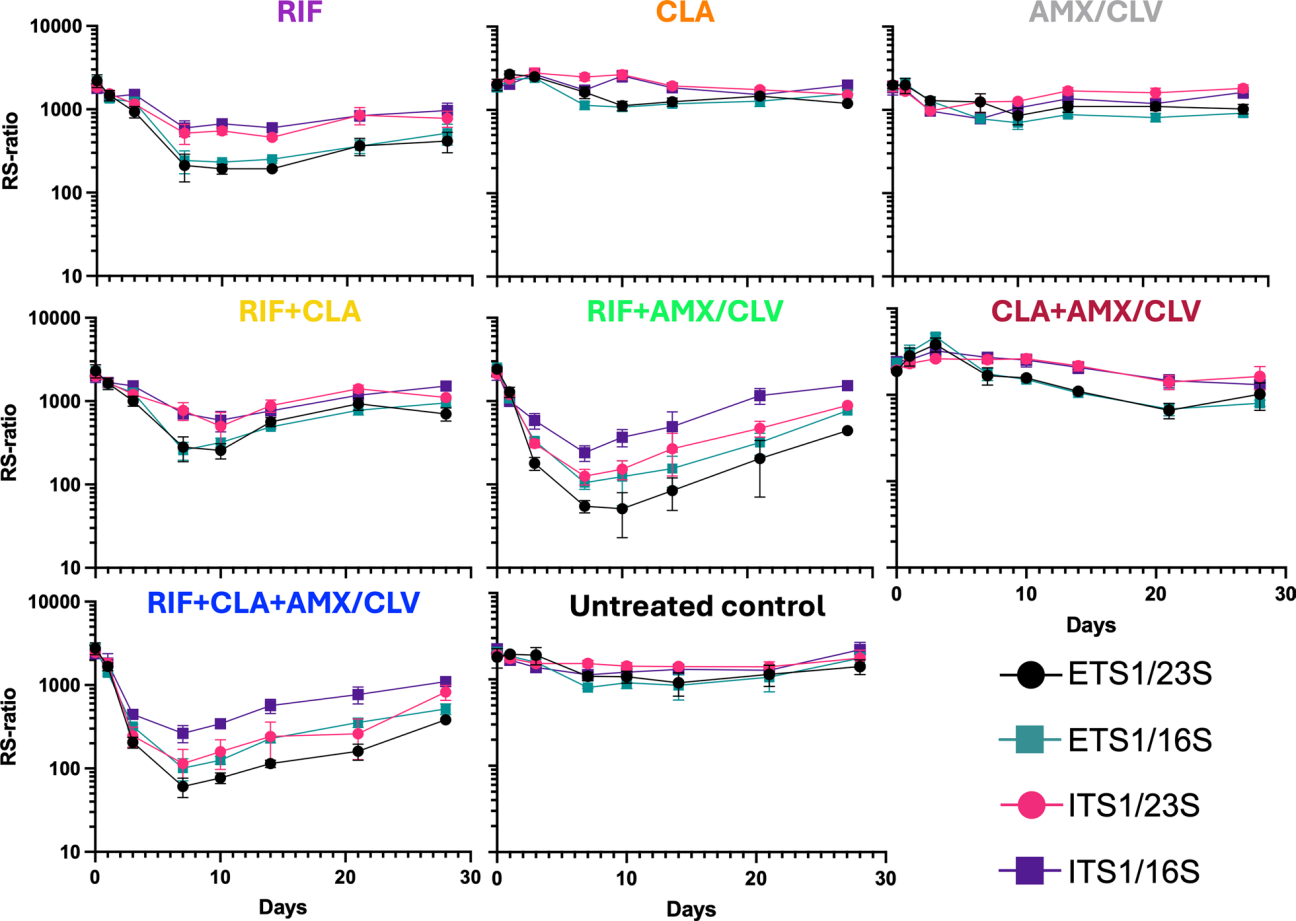
Comparison of four diverse RS-ratio determinations in *Mycobacterium ulcerans* exposed to rifampicin, clarithromycin, and amoxicillin/clavulanate combinations*.* Comparison of the diverse RS-ratio determinations using the different possible pairs of targets ETS1/23S, ITS1/23S, ETS1/16S, and ITS1/16S in *Mul* samples. Values were calculated as: copies of ETS1 or ITS1 divided by copies of 23S or 16S and multiplied by 10^4^. RIF was used at 0.025 µg/ml (1/2 × MIC), CLA at 0.0625 µg/ml (1 × MIC), and AMX at 0.125 µg/ml (1 × MIC in the presence of CLV). CLV was added at a fixed 5 µg/ml concentration. RS-ratio values correspond to a single biological replicate, where data points and error bars represent the mean and standard deviation of technical replicates (2–3) using different cDNA dilutions. *RIF* rifampicin, *CLA* clarithromycin, *AMX/CLV* amoxicillin/clavulanate, *ETS1* external transcribed spacer 1, *ITS1* internal transcribed spacer 1, *RS-ratio* rRNA synthesis ratio, *MIC* Minimum inhibitory concentration

**Fig. 4:**
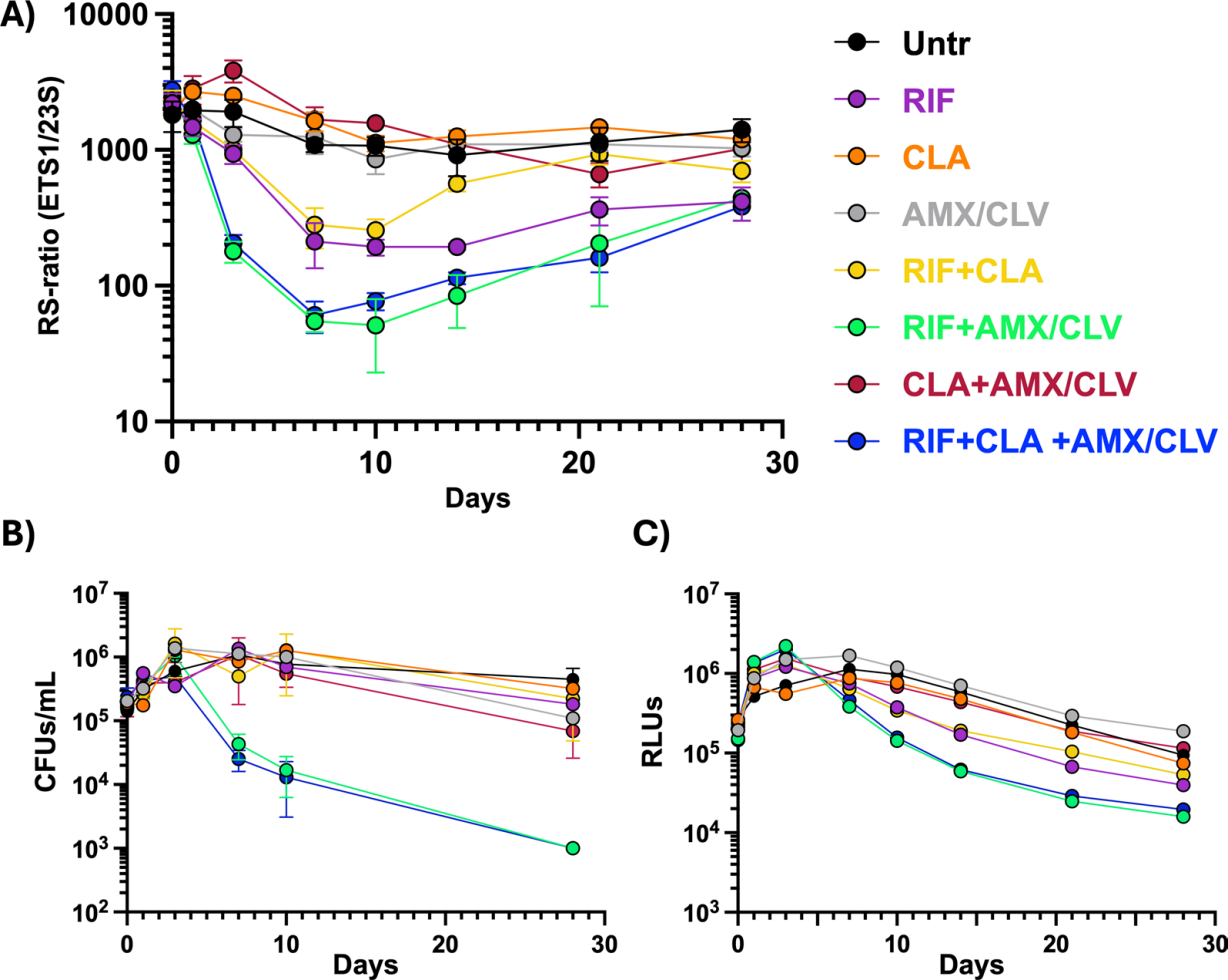
In vitro RS-ratio kinetics of rifampicin, clarithromycin, and amoxicillin/clavulanate combinations against *Mycobacterium ulcerans* in different biomarkers*.* The activity of different drugs alone, in pair-wise and triple combination, was evaluated in time-kill assays against the *Mul* ITM 000932 isolate by **A** RS-ratio (ETS1/23S, copies of ETS1 divided by copies of 23S and multiplied by 10^4^), and compared with the biomarkers previously published by Sáez et al. [4], like **B** colony-forming units, and **C** luminescence readouts. RIF was used at 0.025 µg/ml (1/2 × MIC), CLA at 0.0625 µg/ml (1 × MIC), and AMX at 0.125 µg/ml (1 × MIC in the presence of CLV). CLV was added at a fixed 5 µg/ml concentration. RS-ratio values correspond to a single biological replicate, where data points and error bars represent the mean and standard deviation of technical replicates (2–3) using different cDNA dilutions. *Untr* untreated growth control, *RIF* rifampicin, *CLA* clarithromycin, *AMX/CLV* amoxicillin/clavulanate, *CFU* Colony Forming Units, *RLU* Relative Light Units, *ETS1* external transcribed spacer 1, *RS-ratio* rRNA synthesis ratio, *MIC* Minimum inhibitory concentration

Four distinct RS-ratios were compared employing combinations of a single precursor rRNA (ETS1 or ITS1) and total rRNA (23S or 16S) (Fig. 3). All pairs demonstrated comparable trends across groups. In the conditions where the RS-ratio barely changed over time, such as untreated control, CLA, and AMX/CLV, the different pairs tested (ETS1/23S, ITS1/23S, ETS1/16S, and ITS1/16S) mostly overlapped; however, different dynamic ranges were observed in the conditions in which drug combinations induced a reduction of the RS-ratio. Here, ITS1/16S and ITS1/23S pairs had the lower change, while ETS1/23S and ETS1/16S pairs showed higher change, having the strongest dynamic range the ETS1/23S pair. Thus, the ETS1/23S pair was selected for further comparisons with other biomarkers (Fig. 4).

### RS-ratio kinetics reveal differential metabolic responses of *M. ulcerans* to RIF, CLA, and AMX/CLV combinations

The untreated *Mul* growth control exhibited an elevated RS-ratio (≈2000) (Fig. 4A, black circles), which underwent a slight decrease when the culture reached the stationary growth phase (≈900–1500 at day 7) (Fig. 4B, black circles). Exposure to RIF monotherapy resulted in a substantial reduction in the RS-ratio (≈200) after 7 days of incubation, with a slight recovery at relatively low levels (≈400) until the end of the 28-day assay (Fig. 4A, purple circles). Conversely, CLA monotherapy led to a marginal increasing effect (≈3000) on the RS-ratio (Fig. 4A, orange circles), while AMX/CLV did not significantly affect the RS-ratio (Fig. 4A, grey circles). The combination of CLA + AMX/CLV led to an increasing effect in the RS-ratio levels (≈4000) at day 3 (Fig. 4A, red circles) that dropped close to levels of the untreated growth control after day 7. On the contrary, RIF-containing combos consistently induced a decrease in the RS-ratio, though the intensity and kinetics of this effect appear to be dependent upon the accompanying drug. The RIF + CLA combo reduced the RS-ratio to levels comparable to those attained with RIF monotherapy (≈250) at days 7 and 10 (Fig. 4A, yellow circles). However, after day 14 of incubation, the RS-ratio of *Mul* treated with RIF + CLA showed a recovery to similar levels to the untreated control (≈700–900) that was stronger and faster than the slight increase observed in *Mul* treated with RIF monotherapy. In contrast, both the RIF + AMX/CLV combination and the triple combination (RIF + CLA + AMX/CLV) (Fig. 4A, green and blue circles, respectively) induced the fastest and most potent decreases of the RS-ratio of the conditions tested. A visible reduction (≈200) was already evident at day 3, reaching the maximal inhibition (≈50–60) between days 7 and 10. After day 14, the RS-ratio began to recover (≈400), but the levels of the untreated sample were not reached even at day 28. Importantly, this recovery was not identified by CFUs or luminescence biomarkers (Fig. 4B and C).

## Discussion

This study has investigated for the first time the implementation of the RS-ratio biomarker to assess the in vitro activity of drugs against *Mul*. To this end, four sequences of the rRNA operon of *Mul* were targeted using a primer–probe set previously described [8] and three newly designed primer–probe sets. The implementation of a single multiplexed ddPCR assay confirms the specificity of the different primer–probe sets. Although we used only two closely related mycobacteria (*Mtb* and *Msm*) as internal controls for specificity, our results support previous reports for the 16S rRNA *Mul* set [8]. In silico alignment using NCBI BlastN predicted potential cross-amplification only with *M. marinum* due to high sequence identity in the rRNA operon region. While both species caused skin lesions, only those in early stages can appear similar, with clearly different clinical signs and epidemiology. While our work is currently circumscribed to in vitro drug activity evaluations, testing against a panel of potential BU-relevant skin-coinfection species would be beneficial to provide a more comprehensive validation of the primers and probes in clinical samples.

Additionally, we tested various combinations of precursor-to-total rRNA ratios using a multiplexed ddPCR assay, and we observed that the ETS1/23S rRNA pair demonstrated the highest dynamic range (Fig. 3). The observed variations in the dynamic range of the distinct RS-ratio pairs in response to different treatments may reflect both the direct impact on precursor rRNA transcription and the effect on precursor rRNA maturation pathways. A thorough investigation into the underlying causes of these dynamic differences is beyond the scope of this study. However, these findings underscore the importance of empirically exploring various precursor/total rRNA pairs before implementing the RS-ratio biomarker for a new organism.

A substantial reduction in the RS-ratio was observed in *Mul* treated with RIF monotherapy, despite RIF being purposely added at half of its MIC. This effect was not observed with CLA and AMX/CLV, which were dosed at their respective 1 × MIC values. This observed behavior of the RS-ratio in response to different drugs could be explained by the mechanism of action of each drug. RIF, a cornerstone sterilizing drug in the treatment of *Mtb* and other mycobacterial infections, inhibits transcription by binding to the DNA-dependent RNA polymerase ß-subunit, encoded by the *rpoB* gene [9–11]. In accordance with prior observations in *Mtb* [7, 12], RIF reduced the RS-ratio of *Mul*, likely by directly inhibiting the transcription of precursor-rRNAs. Although there are no extant reports on the effect of CLA or AMX/CLV on the RS-ratio of *Mtb*, our findings are also consistent with documented observed effects of other drugs with a similar mechanism of action in *Mtb*. The macrolide CLA inhibits protein translation of bacterial mRNAs by binding to the 23S rRNA in the 50S subunit of the ribosome [13]. As previously documented, pharmaceutical agents that target translation by binding to the ribosome, such as aminoglycosides (streptomycin) or oxazolidinones (linezolid) [7, 12], cause a modest initial increase in *Mtb* RS-ratio. We observe a similar pattern with CLA against *Mul*. This might represent a potential bacterial response to overcome the inhibition of ribosomal activity. AMX, a semisynthetic penicillin derivative from the ß-lactam family of antibiotics, exerts its antimicrobial effect by inhibiting bacterial cell wall synthesis, thereby preventing peptidoglycan cross-linking by transpeptidase proteins [14]. However, its activity against mycobacteria is hindered due to the constitutive expression of BlaC (a ß-lactamase in mycobacteria), which is also present in *Mul* [15]. Nevertheless, this natural resistance mechanism can be counteracted by the co-administration of CLV, a ß-lactamase inhibitor [3, 16]. Our findings with AMX/CLV are consistent with previous reports showing that other cell wall synthesis inhibitors, such as isoniazid and ethambutol, have a minimal impact on reducing the RS-ratio of *Mtb* even at concentrations several times above their MIC, despite targeting different pathways in cell wall biosynthesis [7, 12].

Our data on the activity of the different combinations against *Mul* using the RS-ratio (Fig. 4A) support previous findings reported by Saez et al*.* [4]. RIF + AMX/CLV-containing combinations induced the most pronounced reductions in RS-ratio and were also identified as the most potent regimens by other biomarkers **(**Fig. 4B and C). This observation is consistent with the notion that the RS-ratio functions as a more expeditious biomarker of sterilizing drug activity. As reported in *Mtb* [7], stronger reductions in the RS-ratio appear to correlate with enhanced sterilizing effects, a proxy for improved treatment outcomes even with shorter treatments. This finding is in accordance with the ongoing clinical trials aiming to shorten BU treatment from 8 to 4 weeks by the inclusion of AMX/CLV to the WHO RIF + CLA recommended treatment [5, 6].

In the context of the in vitro TKA samples used to implement the RS-ratio in *Mul* [4], drugs were administered only at the onset of the experiment at sub-optimal concentrations. Although this approach does not reproduce dynamic drug exposure, it also does not imply constant or stable drug concentrations throughout the experiment. Instead, drug concentrations are expected to change over time according to the compound-specific half-lives in the in vitro assay media, which are important determinants of drug effect. For instance, RIF has an in vitro half-life of approximately seven days, with about 75% degradation after 14 days of incubation in 7H9 medium at 37 °C [17–19]. A comparable or even shorter media half-life (14.2 h) has been documented for CLA [17], while the half-life of AMX fluctuates extensively (from 27.4 h to 10 days) contingent on media conditions [20–23]. Indeed, a slight recovery in the RS-ratio was observed after 10 days of incubation in the different RIF-containing treatments, including those containing AMX/CLV, even as bacterial burden levels continued to decline (Fig. 4B and C) [4]. This phenomenon may be attributed to the initial sub-optimal concentration of RIF (1/2 × MIC) and its degradation over time, likely limiting the drug efficacy and enabling bacterial metabolic recovery. In a preceding TKA study on *Mtb*, even concentrations of RIF 20 × MIC were unable to prevent bacterial regrowth at endpoint due to RIF degradation in the assay medium [19].

A detailed comparison between conditions of RIF monotherapy and RIF-CLA combination reveals a faster recovery of the RS-ratio in the presence of CLA after day 10 (Fig. 4A). This is in line with previous observations showing that CLA monotherapy results in a modest increase in the RS-ratio. Given the similar drug stability, the observed accelerated recovery is likely due to the differential initial dose and the opposite effects of CLA and RIF on the RS-ratio. This interaction likely enables CLA to impair the RS-ratio reducing effect of RIF as its concentrations decline over time. This increase in the RS-ratio anticipates a potential antagonistic effect observed after day 21 with other biomarkers; for example, luminescence from the RIF-CLA combination is reduced more slowly than that from RIF monotherapy (Fig. 4C) [4].

**Fig. 5:**
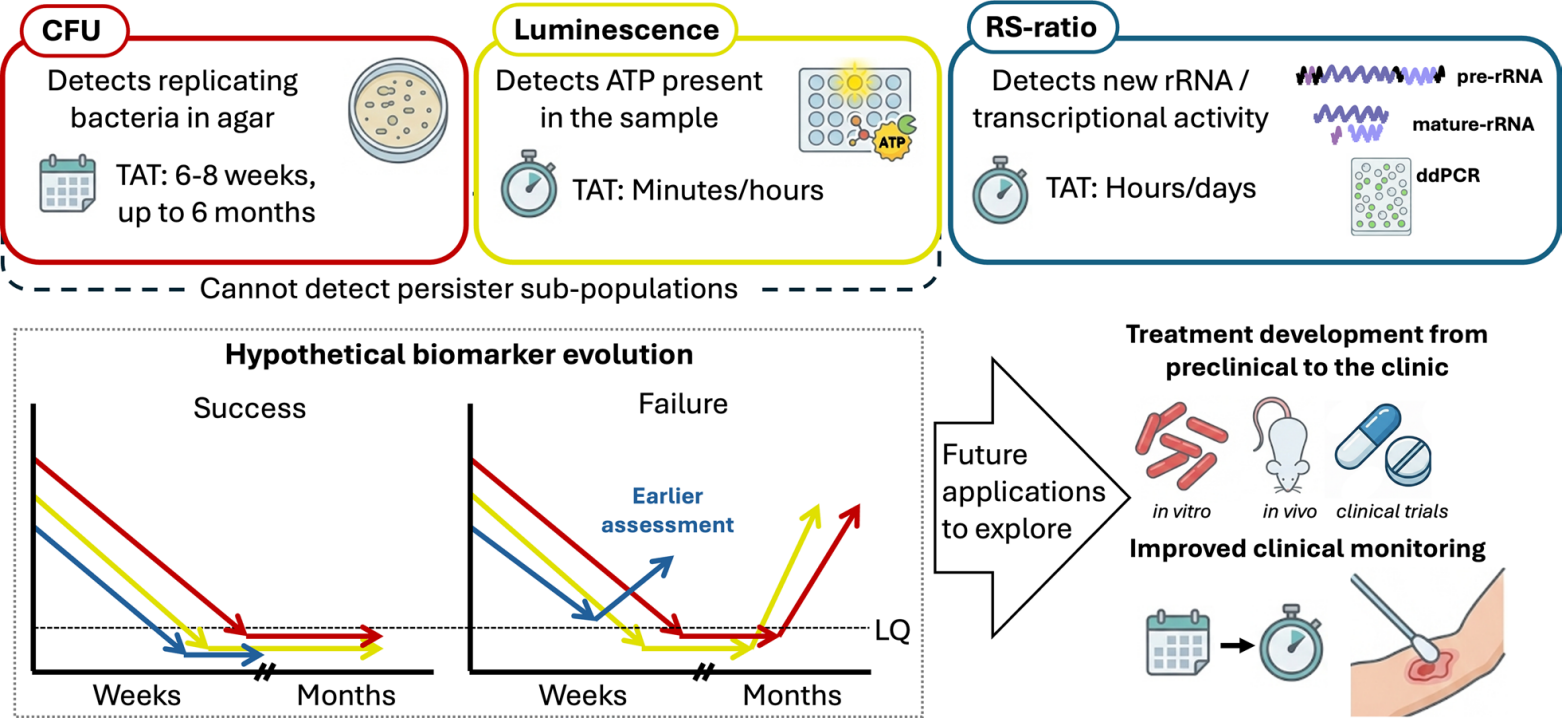
Schematic comparison of the different biomarkers for *Mycobacterium ulcerans* treatment evaluation. Colony-forming units (CFU; red boxes/arrows) detect bacterial subpopulations capable of replicating on agar, yielding results with long turnaround times (TATs) of weeks or months for *Mul*. Luminescence (yellow boxes/arrows) via BacTiter-Glo measures ATP levels present in the sample through an enzymatic reaction that releases light, providing results in short TATs of minutes or hours. The RS-ratio (blue boxes/arrows) measures the transcription and biogenesis of rRNA, providing results in short-to-modest TATs of hours or days, including all sample processing steps. Conceptually, these biomarkers should hypothetically exhibit different dynamics during treatment assessment in *Mul*: even if they drop below quantification limits, confirming success via CFU or luminescence requires extended follow-up for several months to confirm that no *Mul* re-growth occurs, due to their limitation of missing the activity of bacterial persisters. Furthermore, the long TAT of CFUs for *Mul* further delays the confirmation of results. In contrast, the RS-ratio may anticipate treatment success or failure by detecting ongoing rRNA synthesis. The RS-ratio could be applied to evaluate new drug regimens in preclinical and clinical studies, and it could even serve as a monitoring biomarker to anticipate patient outcomes. *LQ* limit of quantification, *ddPCR* droplet digital PCR, *RS-ratio* rRNA synthesis ratio

The addition of AMX/CLV to RIF-containing regimens led to the most significant reductions in the RS-ratio (Fig. 4A) in line with observations using other biomarkers (Fig. 4B and C). In a previous study [19], combining RIF with ß-lactams (in particular, with first-generation cephalosporins) prevented bacterial regrowth at endpoint, despite substantial drug degradation of both compounds. While no correlation with the RS-ratio was performed at that time, combining those findings with the present data provides the first insights into the sterilizing effect of RIF and ß-lactams (i.e., AMX/CLV) combinations against mycobacterial species. RS-ratio recovery patterns were observed in this retrospective study; this is because the experimental design used by Saez et al*.* [4] was only powered to identify synergistic interactions (i.e., drugs were evaluated at sub-optimal concentrations) and not to assess the full capacity of drugs alone or in combination, which would have required higher doses, such as in the above-mentioned study [19]. Consequently, the observed RS-ratio recovery patterns suggest the existence of a residual bacterial subpopulation that survived the initial treatments. This sub-population would likely resume metabolic activity once drug concentrations dropped below effective levels, suggesting that culture regrowth might occur if longer TKA times were provided. As such, the RS-ratio potentially functions as an early monitoring biomarker, capable not only of ranking potential sterilizing drug combinations but also of detecting the early onset of metabolic bacterial reactivation and regrowth (Fig. 5). Further investigations should be conducted to confirm whether the RS-ratio could also be used to detect the presence of bacterial resistance or tolerance. Interestingly, even at sub-optimal concentrations, the combination of RIF + AMX/CLV was more effective than either drug alone. This supports the synergistic interaction and treatment-shortening potential of this novel regimen.

While administering single doses of suboptimal concentrations of different drugs helps to understand in vitro the pattern of synergistic drug interactions against *Mul*, it does not accurately represent in vivo and clinical exposures needed for translational efforts. Optimal drug concentrations will yield enhanced reductions of the RS-ratio in *Mul*, in accordance with documented evidence of RIF dose–response effects against *Mtb,* both in vivo [7] and in vitro [12]. Therefore, there is compelling rationale for implementing the RS-ratio analysis in in vivo models, when necessary, and in more complex in vitro models, such as the Hollow Fibre System (HFS). The HFS provides dynamic measures of pharmacokinetic and pharmacodynamic relationships of antimicrobial treatments against a pathogen of interest; it was endorsed by the European Medicines Agency for the study of *Mtb* drug treatments [24], and it is currently under further optimization [25].

This study has several limitations. Our analyses were conducted retrospectively using previously generated in vitro TKA samples under static culture conditions and suboptimal drug concentrations. The experiments were performed using a single clinical isolate (*Mul* ITM 000932), which may not reflect the biological diversity of *Mul* in endemic BU settings. In addition, TKA conditions do not reproduce the pharmacokinetic profiles encountered in patients. Therefore, our results should be interpreted as exploratory and hypothesis-generating, pending validation in additional isolates and in more complex experimental and clinical settings.

Overall, we have described for the first time the implementation of the RS-ratio biomarker for BU research, measuring bacterial metabolic activity and growth dynamics together with in vitro drug activity against *Mul*. These activities align with the WHO-recommended key areas of development of rapid diagnostic tests for BU that can be used at the primary health care level and the development of shorter BU treatments [1]. The RS-ratio offered novel insights into the impact of pharmaceutical agents on persistent bacterial activity, a capability that conventional gold standard methodologies of bacterial load quantification by CFU enumeration do not possess. In addition, research in *Mtb* has demonstrated a link of substantial reductions in the RS-ratio with treatment-shortening properties of novel regimens. Our data support this association for the RIF-AMX/CLV-based combinations currently under evaluation in BLMs4BU clinical trials [5, 6]. This finding suggests that the RS-ratio biomarker has the potential to inform the development of more efficacious and efficient regimens for the treatment of BU, TB, and even other infectious diseases. In conclusion, while our findings are in vitro and primarily hypothesis-generating, they provide a compelling rationale for next translational steps and justify further investigation into the use of the RS-ratio as a novel biomarker in complex in vitro platforms (such as the HFS), animal models, and clinical settings for the monitoring of BU treatment shortening effectiveness.

## Data Availability

All data generated or analyzed relating to this study are presented within this published article.

## Abbreviations

16S: 16S rRNA
23S: 23S rRNA
AMX: Amoxicillin
BU: Buruli ulcer
C-: Negative control
cDNA: Complementary DNA
CFUs: Colony-forming units
CLA: Clarithromycin
CLV: Clavulanate
Cy5: Cyanine-5
ddPCR: Droplet digital PCR
ETS: External transcribed spacer
FAM: 6-Carboxyfluorescein
Fw: Forward
HEX: Hexachlorofluorescein
HFS: Hollow fibre system
ITS: Internal transcribed spacer
LQ: Limit of quantification
MIC: Minimum inhibitory concentration
Msm: Mycobacterium smegmatis
Mtb: Mycobacterium tuberculosis
Mul: Mycobacterium ulcerans
NTD: Neglected tropical diseases
RIF: Rifampicin
RLU: Relative light units
ROX: Rhodamine X
rRNAs: Ribosomal RNAs
RS-ratio: Ribosomal RNA synthesis ratio
Rv: Reverse
TATs: Turnaround time
TB: Tuberculosis
TKA: Time-kill assay
Untr: Untreated growth control
WHO: World Health Organization
